# Supplementary material for: A Relational Formulation of Quantum Mechanics
Source: Sci Rep. 2018 Sep 6;8:13305. doi: 10.1038/s41598-018-31481-8 (PMC6127378; doi:10.1038/s41598-018-31481-8)
Supplement: Supplementary file 1 — Supplemental Footnotes [file 41598_2018_31481_MOESM1_ESM.pdf]

A Relational Formulation of Quantum Mechanics
Supplementary Information

Jianhao M. Yang*
Qualcomm, San Diego, CA 92121, USA

This supplementary information contains additional notes for some of the statements in the article.

1. Traditional quantum mechanics does not provide a theoretical description of Process 1. In the Copenhagen Interpretation, this is considered as the "collapse" of the wave function into an eigenstate of the measured observable. The nature of this wave function collapse has been debated over many decades.

2. The bidirectional process does not necessarily imply two sequential steps. Instead, it is better to be understood as two aspects of a complete process in a measurement event. We can use a classical probability problem to analogize this. Suppose tossing a special coin gets a face up with probability of p. If a certain process requires tossing two such coins in the same time, we ask what is the probability of a process that resulting in one coin facing up and one coin facing down. The answer is $p(1-p)$. The difference between this analogy and a quantum measurement is that in a quantum measurement, each aspect of the process itself is not necessarily assigned a real non-negative number.

3. In other words, a probabilistic quantity is a non-negative real number only when it is associated with an actual physical measurement. Such a requirement does not need to be true for probabilistic quantity associated with an incomplete, one-way process. A similar argument can be found in ref.[18]. There is also a temptation to express the relational variable as $Q^{A\to S}(|a_j\rangle \mid |s_i\rangle)$, making it looks like a conditional probability quantity. However, we choose the expression $Q^{A\to S}(|a_j\rangle \cap |s_i\rangle)$ because it better represents a relational quantity for a joint event.

4. When the correlation between S and A are established, both systems are effectively measuring each other (see similar remark in ref. [10]). Change occurs either in S or in A will be reflected by the relational matrix element. But there should not have a preference of considering S or A as a measuring system.

5. The situation when inference information is available is discussed in the next subsection. In probability theory, it is crucial not to under-count or over-count applicable alternatives when calculating probability. When a quantum system is in a superposition state, although each eigenvector is labeled with a different ket, each ket should be considered indistinguishable for counting purpose because there is no information to determine exactly which ket the system is in. It is an under-count if only considering $|a_j\rangle \to |s_i\rangle \to |a_j\rangle$. There is similar example in statistical physics. When counting the number of microscopic states of an ensemble consisting of identical particles, one strategy is to first over-count by assuming the particles are distinguishable, then divide the counting result by a factor to offset the over-counting.

6. From Postulate 2e, the probability of finding the composite system $S + A$ in an eigenvector $|m\rangle$ is $p_m = |\sum_n R'_{mn}|^2$. From Postulate 4, $|m\rangle$ can be rewritten to be $|s_i\rangle|a_j\rangle$ by renumbering index m to i, j since m is defined in the Hilbert space $\mathcal{H}_S \otimes \mathcal{H}_A$. Therefore p_m is the probability for the combined events $|s_i\rangle$ for S and $|a_j\rangle$ for A, i.e., $p_m = p_{ij}$. But $p_{ij} = |R_{ij}|^2$ so that $|\sum_n R'_{mn}|^2 = |R_{ij}|^2$. This gives $\sum_n R'_{mn} = e^{i\phi} R_{ij}$ where $e^{i\phi}$ is an unimportant phase factor.

7. This property is clearer when O is the representation of a unitary operator. In that case, operator $\hat{O}(t - t_0) = e^{-i\hat{H}(t-t_0)/\hbar}$ where $\hat{H}$ is a Hermitian operator. Reverting the parameter of time gives $\hat{O}(t_0 - t) = e^{-i\hat{H}(t_0-t)/\hbar} = e^{i\hat{H}(t-t_0)/\hbar} = O^\dagger(t - t_0)$.

8. Mathematically, the Specht's Theorem and its improved version Pearcy's Theorem give the necessary and sufficient conditions for two matrices to be similar[25]. This allows one to determine if $\rho(t)$ and $\rho(0)$ are unitary similar matrices. However, how such condition is related to whether $Q(t)$ and $O(t)$ are unitary matrices is not obvious. It requires further investigation.

9. It is easier to realize the non-commutation if using the matrix representation of Eq. (40): $i\hbar(dR(t)/dt) = H_S R(t) + R(t)H_A^T$. Since R is a $N \times M$ matrix while H_A^T is a $M \times M$ matrix, matrix multiplication $H_A^T \times R$ is even not possible when $N \neq M$.

* jianhao.yang@alumni.utoronto.ca
